# Mixed Exposure to Underground Air Pollutants and Metabolic Syndrome in Coal Miners: A Cross-Sectional Study Integrating Multi-Pollutant Models and Urinary Metabolomics

**DOI:** 10.3390/toxics14090746

**Published:** 2026-08-24

**Authors:** Jia Wang, Shuying Chen, Chenyi Wang, Wenwen Li, Yuanjie Zou, Fenglin Zhu, Min Mu

**Affiliations:** 1 The First Hospital of Anhui University of Science and Technology, Huainan 232001, China; wangjia@aust.edu.cn; 2 School of Public Health, Anhui University of Science and Technology, Huainan 232001, China; chenshuying13543862418@163.com (S.C.); wangchenyi1029@163.com (C.W.); liwenwen17732435633@163.com (W.L.);; 3 Key Laboratory of Industrial Dust Control and Occupational Health of the Ministry of Education, Anhui University of Science and Technology, Huainan 232001, China

**Keywords:** mines, air pollutant exposure, metabolic syndrome, metabolomics

## Abstract

To investigate the associations between single and mixed exposure to air pollutants in underground coal mine environments and the risk of metabolic syndrome (MetS) among workers, we conducted a cross-sectional study. Multivariable logistic regression, Bayesian kernel machine regression (BKMR), and quantile-based g-computation (Qgcomp) were used to evaluate the joint effects of mixed pollutant exposures and to identify the major contributing components. In addition, untargeted urinary metabolomics analysis was performed to explore the potential biological mechanisms. After adjusting for confounding factors, logistic regression analysis revealed that exposure to coal dust (CD), carbon monoxide (CO), carbon dioxide (CO_2_), and nitrogen dioxide (NO_2_) was associated with an increased risk of MetS. Furthermore, BKMR and Qgcomp models consistently indicated a significant positive association between mixed pollutant exposure and MetS risk, with CD and NO_2_ identified as the primary components with the strongest statistical contribution. CD exposure was mainly associated with central obesity and hypertension, whereas NO_2_ exposure was primarily linked to elevated blood glucose and triglyceride levels. MetS patients exhibited significant alterations in urinary metabolic profiles, with more than 113 differentially abundant metabolites identified. Notably, leukotrienes were positively correlated with NO_2_ exposure, while acylcarnitines were associated with CD exposure. Pathway enrichment analysis indicated significant disturbances in pyrimidine and arachidonic acid metabolism. These findings provide important evidence for developing comprehensive occupational health strategies in mining environments.

## 1. Introduction

Metabolic syndrome (MetS) is a clinical condition characterized by central obesity, elevated blood pressure, impaired fasting glucose (or insulin resistance), and abnormal lipid levels (including elevated triglycerides and/or reduced high-density lipoprotein cholesterol levels) [1,2]. It is a well-established major risk factor for cardiovascular disease, type 2 diabetes, stroke, non-alcoholic fatty liver disease, and certain types of cancers [3]. MetS is widely recognized as a major global public health challenge [4]. Current estimates indicate that approximately one in four adults worldwide meets the diagnostic criteria for MetS, and its prevalence has increased substantially over the past three decades [5]. For instance, between 2000 and 2023, the global age-standardized prevalence of MetS increased from 14.7% to 31.0% among women, and from 9.0% to 25.7% among men [6]. Among U.S. adults, the age-standardized prevalence rose from 27.9% during 1999–2006 to 41.8% during 2017–2018 [7]. Similarly, national surveys in China reported an increase in prevalence from 13.7% to 31.1% between 2001 and 2017 [8].

Traditional risk factors for MetS include advancing age, low socioeconomic status, physical inactivity, unhealthy dietary patterns, and genetic predisposition [9,10,11,12]. However, a growing body of evidence highlights the important role of environmental pollutants in the development of MetS [11,13,14,15]. Notably, long-term exposure to ambient air pollution has been consistently associated with an increased incidence and prevalence of MetS. This association may be mediated through several biological mechanisms, including systemic inflammation, oxidative stress, endothelial dysfunction, and gut microbiota dysbiosis [4,11]. Robust epidemiological evidence linked MetS to exposure to major air pollutants, including fine and coarse particulate matter (PM_1_, PM_2.5_, and PM_10_), NO_2_, sulfur dioxide (SO_2_), and ozone (O_3_) [16,17,18,19,20]. Geographically, these associations have been replicated across diverse high- and middle-income countries. Positive associations between particulate matter exposure and adverse metabolic outcomes have been reported in Germany, Switzerland, the United States, and South Korea [21,22,23]. Similar findings have also been observed in China, where multiple population-based studies have confirmed the adverse metabolic effects of air pollution [13,24]. For example, a nationally representative cohort study of Chinese adults aged ≥ 45 years demonstrated that long-term exposure to PM_2.5_ significantly increased the risk of both MetS and its core components, particularly central obesity and dyslipidemia. The observed associations were substantially stronger among older adults and urban residents [25]. Importantly, the adverse effects of air pollution are not limited to adults. Chronic exposure to PM_2.5_, PM_10_, and NO_2_ has also been associated with a higher prevalence of MetS among children and adolescents, with sex-stratified analyses indicating stronger associations in boys [19].

However, most current research on MetS has focused primarily on community-based populations, with relatively few studies examining the relationship between occupational environmental exposures and MetS. Existing occupational cohort studies suggest that long-term exposure to heavy metals, dust, PM_10_, SO_2_, and CO may significantly increase the risk of MetS [26,27,28]. For instance, a study involving middle-aged and older employees at the Electricity Generating Authority of Thailand found that chronic occupational exposure to PM_10_, SO_2_, and CO was associated with a higher prevalence of MetS [29]. Similarly, a 15-year prospective cohort study of flour mill workers in Iran reported that long-term exposure to flour dust was associated with an increased risk of hypertension and hypertriglyceridemia, with stronger associations observed among workers with longer daily shifts or rotating shift schedules [30]. Industries such as mining, metallurgy, and chemicals are known for high concentrations of complex air pollutants, making them priority settings for occupational health research on hazardous exposures [31,32,33]. Underground coal mining presents unique challenges due to confined working environments, complex ventilation systems, and persistent exposure to high levels of occupational dust (primarily a mixture of crystalline silica and metal-containing dust). Diesel-powered equipment further contributes nitrogen oxides (NO_x_, especially NO_2_) and CO, while CO_2_ is generated during blasting operations and rock oxidation [34,35,36,37]. Collectively, these agents constitute a dynamic and heterogeneous mixture of airborne pollutants, including both aerosols and gases. To date, most occupational health studies have primarily focused on the association between dust exposure and organ-specific diseases, particularly pneumoconiosis [38]. In contrast, the potential contribution of complex pollutant mixtures to the development of systemic, multi-organ conditions such as MetS remains insufficiently investigated.

This study integrates occupational epidemiology and untargeted metabolomics to investigate the associations between individual and mixed exposures to major underground coal mine air pollutants (including respirable CD, CO, CO_2_, NO, and NO_2_) and MetS among coal miners. Multivariable logistic regression models will first assess the independent associations between each pollutant and MetS. Subsequently, the complementary mixture-analysis methods, including BKMR and Qgcomp, will be used to evaluate the joint effects of pollutant mixtures and identify the major contributors to MetS risk. Finally, untargeted urinary metabolomics will be performed to identify potential biomarkers and disrupted metabolic pathways associated with environmental exposures. Together, these analyses will improve understanding of the health effects and potential biological mechanisms underlying occupational air pollutant exposure and MetS in underground coal miners.

## 2. Materials and Methods

### 2.1. Study Design and Population

A cross-sectional study was conducted among 1689 male miners employed at a mining enterprise in northern China (21 July–21 August 2023). Sociodemographic characteristics, occupational history, and lifestyle information were collected from all participants using a standardized questionnaire. In addition, physical examinations, including measurements of height, weight, waist circumference, hip circumference, and blood pressure, were performed by medical staff at a local health examination center. Fasting venous blood samples were also collected to assess biochemical parameters, including fasting glucose (FG), triglycerides (TGs), and high-density lipoprotein cholesterol (HDL-C).

The study protocol was approved by the Medical Ethics Review Committee of the First Affiliated Hospital of Anhui University of Science and Technology (Ethics Approval No.: 2023-KY-112-001, Date: 1 July 2023), and written informed consent was obtained from all participants.

### 2.2. Inclusion and Exclusion Criteria

Inclusion Criteria: (1) Male coal miners with a history of underground environmental exposure; (2) no previous history of severe organic diseases; (3) completion of routine health examination procedures.

Exclusion Criteria: (1) Individuals with severe cardiovascular, cerebrovascular, hepatic, renal, or other major malignant diseases; (2) individuals with severe mental disorders or other conditions that interfered with compliance with medical examinations; (3) individuals with a history of surgery, radiotherapy, or chemotherapy for malignant tumors; (4) individuals with severe infectious diseases affecting multiple organ systems; (5) individuals with missing or incomplete data related to metabolic syndrome (MetS) indicators.

### 2.3. Definition of MetS

MetS was defined according to the International Diabetes Federation (IDF) criteria for Asian populations. The diagnostic components included: (1) elevated triglyceride (TG) levels (≥1.7 mmol/L [150 mg/dL]); (2) reduced high-density lipoprotein cholesterol (HDL-C) levels (<1.0 mmol/L [40 mg/dL] in men); (3) elevated blood pressure, defined as systolic blood pressure (SBP) ≥ 130 mmHg and/or diastolic blood pressure (DBP) ≥ 85 mmHg; (4) elevated fasting glucose (FG) levels (≥5.6 mmol/L [100 mg/dL]); and (5) central obesity, defined as waist circumference (WC) ≥ 90 cm in men. Participants meeting at least three of the five criteria were classified as having MetS.

### 2.4. Exposure Assessment

Cumulative exposure to underground air pollutants was quantified using a standardized protocol [38]. Miners wore pre-calibrated personal sampling devices during their shifts, and sampling was conducted over three consecutive workdays to account for intra-week variability in mining activities (e.g., blasting frequency, equipment usage). After each shift, the devices were collected, and exposure durations were cross-verified with shift logs to calculate 8-h time-weighted average (TWA) concentrations. CD was measured by the gravimetric method (filter weighing) using Sensodyne respirable dust samplers (Gilian^®^ 5000, NIOSH-compliant, Sensidyne, LP, Clearwater, FL, USA; accuracy: ±5%, range: 1–100 mg/m^3^). CO and CO_2_ were measured by non-dispersive infrared (NDIR) absorption using portable CO/CO_2_ analyzers (Beijing Huayun, GXH-3011A, Beijing Huayun Analytical Instrument Research Institute Co., Ltd., Beijing, China; accuracy: ±2% full scale, calibrated with NIST-traceable gas). NO_2_ was measured by the electrochemical sensor method using a nitrogen dioxide analyzer (Shenzhen Keernuo, GT-1000-NO_2,_ Shenzhen Keernuo Electronic Technology Co., Ltd., Shenzhen, China; accuracy: ±3% of reading, range: 0–20 ppm). NO was measured by chemiluminescence using a portable NO_x_ analyzer (Wuhan Tianhong, TH-2001H, Wuhan Tianhong Environmental Protection Industry Co., Ltd., Wuhan, China; accuracy: ±2.5% full scale, range: 0–50 ppm).

Based on detailed work histories (job types and durations) and exposure level data TWA, cumulative exposure dose (concentration × time) for each miner was estimated in units of mg/m^3^·year. The pollutants assessed included CD, CO, CO_2_, NO, and NO_2_. Cumulative CD exposure was calculated as the sum of the products of the dust exposure concentration and duration of exposure for each job held by the miner, using the following formula [38]:

$$Cumulative\;Exposure=\sum_{j=1}^{n}\left(C_{j}\;*\;T_{j}\right)$$

where *n* is the total number of job types, j represents a specific job, Cj is the 8-h TWA dust exposure concentration (mg/m^3^) for the j-th job, and Tj is the duration of exposure (years) in the corresponding job. Cumulative exposure doses for the remaining pollutants were calculated using the same approach.

### 2.5. Urinary Metabolomics Analysis

To further explore the urinary metabolomic characteristics of patients with MetS, this study performed a small-sample expanded case–control study based on the original case–control design. Among the 457 MetS participants, uniformly distributed random numbers were generated using SPSS 27.0 statistical software (IBM Corp., Armonk, NY, USA; random seed = 20250613). After ranking participants according to the generated random numbers, 60 individuals were randomly selected as the MetS case group. Subsequently, 60 Non-MetS participants were selected from the original population as the control group using frequency matching based on potential confounding factors, including age, smoking status, and alcohol consumption. Urinary metabolomic analyses were then performed for both groups. Comparisons of baseline characteristics between the MetS and non-MetS groups are presented in Table S1.

First-morning urine samples were collected during physical examinations. After thawing at 4 °C, samples were precipitated, centrifuged, and the supernatants were analyzed via untargeted ultra-high-performance liquid chromatography-tandem mass spectrometry (UHPLC-MS/MS) metabolomics. Pooled quality control (QC) samples were injected periodically, and metabolic features with relative standard deviation (RSD) > 30% in QC were excluded. Following data preprocessing (peak detection, alignment, normalization), principal component analysis (PCA) and supervised partial least squares discriminant analysis (PLS-DA) were performed to visualize global metabolic profiles and group separations. Model validity was assessed by permutation testing (*n* = 200), with R^2^Y and Q^2^ values reported. Differentially abundant metabolites were screened using VIP > 1.0 from the PLS-DA model, followed by Benjamini–Hochberg FDR correction; metabolites with q < 0.14 were considered significant (threshold relaxed to reduce Type II errors given the exploratory design and small subsample, *n* = 200). Metabolite identification was achieved by matching accurate mass and MS/MS fragmentation against HMDB, METLIN, and MassBank, with confidence levels assigned per Metabolomics Standards Initiative (MSI) criteria. Spearman correlation assessed associations between identified metabolites, exposure levels, and clinical parameters. Pathway enrichment was performed using KEGG. All analyses were conducted on the Majorbio platform (Majorbio Cloud 2024, https://www.majorbio.com/).

### 2.6. Covariates

The following covariates were included to account for potential confounding and mediating effects: age (years), education level (below high school, high school or equivalent, above high school), marital status (never married, married), alcohol consumption (never, current or former drinking), smoking status (never, current or former smoker), shift work (yes/no) and sleep quality (good, fair, poor, very poor [requiring medication], or unspecified).

### 2.7. Statistical Analysis

Statistical analyses were performed using R software (version 4.5.2). Descriptive statistics were expressed as frequencies (%) for categorical variables, which were compared using the χ^2^ test; mean ± standard deviation (SD) for normally distributed continuous variables, which were compared using independent-samples *t*-tests; and median (P25, P75) for non-normally distributed variables, which were compared using the Wilcoxon rank-sum test. A two-sided *p* value < 0.05 was considered statistically significant.

Multivariable logistic regression models were used to evaluate the associations between exposure to air pollutants in underground confined spaces and MetS. Pollutants (CD, CO, CO_2_, NO, and NO_2_) were categorized into quartiles, with the lowest quartile (Q1) used as the reference group. Model 1 estimated crude associations; Model 2 was adjusted for age; and Model 3 was additionally adjusted for age, shift work, marital status, education level, sleep quality, smoking, and alcohol consumption. Missing covariate data were classified into a separate “missing” category to minimize data loss. Additionally, we adjusted only for covariates that are not MetS components (such as age, socioeconomic indicators, and lifestyle) to prevent overadjustment for variables directly associated with the outcome.

Two complementary mixture-analysis approaches were used to evaluate the joint effects of co-exposure to five major air pollutants in underground coal mines on MetS and its individual components. (1) BKMR was applied to estimate changes in MetS risk associated with a target pollutant, while the concentrations of the other four pollutants were held constant at their 25th, 50th, and 75th percentiles. The model was fitted using the kmbayes function with 10,000 Markov chain Monte Carlo iterations, and convergence was assessed by inspecting trace plots of β and σ. The overall mixture effect was estimated as the change in the outcome when all exposures simultaneously increased from the 25th to the 75th percentile, with covariates fixed at their median values. Single-pollutant exposure-response functions were estimated by varying one pollutant at a time while holding the other pollutants at their median concentrations, with the 50th percentile used as the reference level. Risk summaries were derived using the Overal lRisk Summaries function with method = “approx” across quantiles from 0.25 to 0.75 in increments of 0.05. In addition, stratified BKMR models were constructed using each MetS component, including TGs, HDL-C, SBP, DBP, FG, and WC, as the outcome variable; (2) Qgcomp was applied to categorize the five pollutants into quartiles (*q* = 4) and to fit a joint marginal structural model for estimating the overall association of the pollutant mixture with the outcome, as well as the relative weights of individual pollutants. The joint odds ratio (OR) and corresponding 95% confidence interval (CI) were estimated using the bootstrap implementation of qgcomp (qgcomp.boot) with 1000 replicates and a random seed of 125. The non-bootstrap implementation (qgcomp.noboot) was used to derive the positive and negative mixture weights for each pollutant. Additionally, stratified qgcomp models were fitted for the MetS component to assess the positive and negative contributions of individual pollutants to specific metabolic indicators.

## 3. Results

### 3.1. Baseline Characteristics of the Study Population

A total of 1689 participants were initially enrolled in this study (Table 1). Of these, 275 were excluded due to missing key variables or a prior diagnosis of severe liver, kidney, or lung diseases, including pneumoconiosis. The final analysis therefore included 1414 participants, of whom 457 (32.3%) were classified in the MetS group and 957 (67.7%) in the non-MetS group. The baseline characteristics of both groups are summarized in Table 1. Compared with the non-MetS group, participants in the MetS group were significantly older (*p* < 0.001) and had larger waist circumferences (*p* < 0.001). Regarding occupational exposures, the median cumulative exposure levels to CD, CO_2_, and NO_2_ were significantly higher in the MetS group than in the non-MetS group (*p* < 0.001). Additionally, the MetS group included a higher proportion of participants with lower educational attainment (high school or below), those engaged in shift work, and those with a history of hypertension, hyperlipidemia, or diabetes, compared with the non-MetS group (*p* < 0.05). No statistically significant differences were observed between the two groups in terms of smoking status, alcohol consumption, or sleep quality (*p* > 0.05).

### 3.2. Association Between Single-Exposure to Five Underground Air Pollutants and Metabolic Syndrome Risk

**Figure 1:**
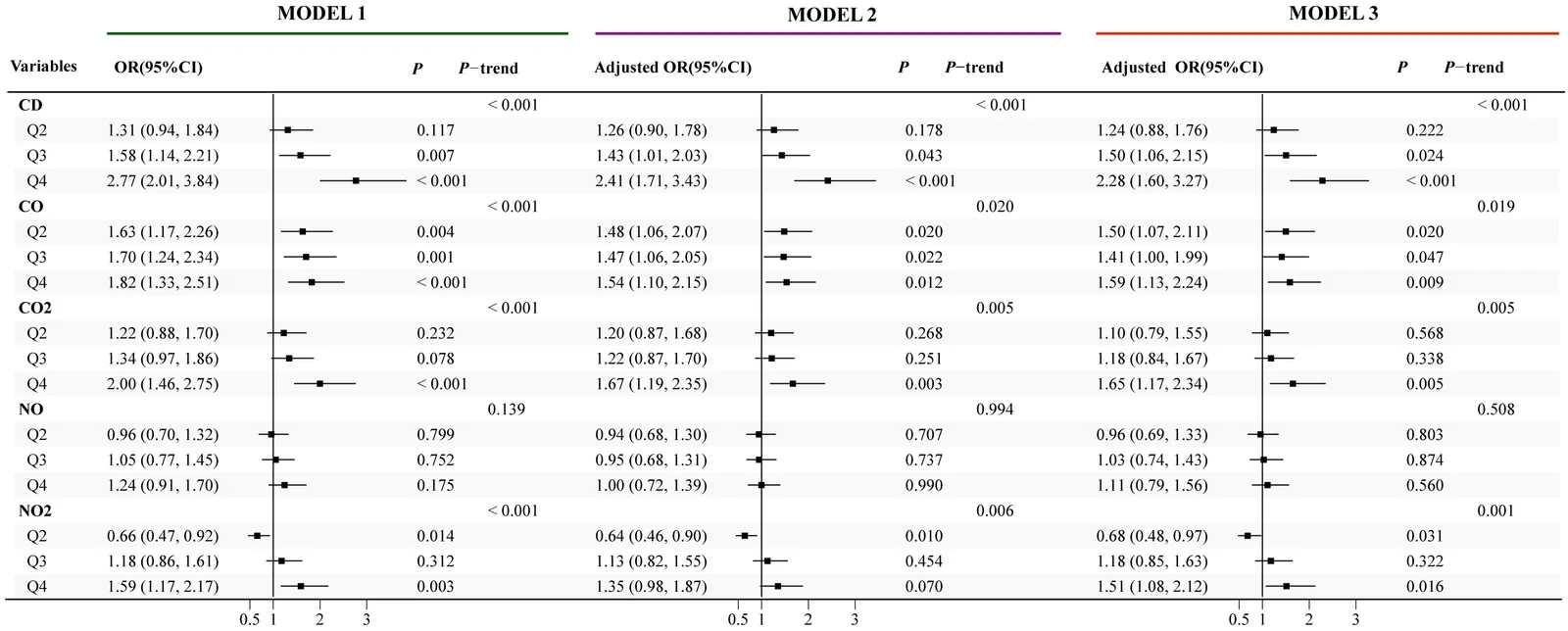
Associations between underground air pollutant concentrations and metabolic syndrome risk in multivariable-adjusted logistic regression models. Model 1: unadjusted; Model 2: adjusted for age; Model 3: adjusted for age, shift work, marital status, education level, sleep quality, smoking, and alcohol consumption.

To examine the associations between individual air pollutants and the risk of MetS, logistic regression models were used. The pollutants were divided into quartiles based on their concentration levels, with the lowest concentration group (first quartile) serving as the reference (Figure 1). In Model1, cumulative exposure to CD, CO_2_, NO_2_, and CO was significantly associated with an increased risk of MetS (*p* < 0.001). In contrast, cumulative exposure to NO showed no significant association. In Model 2, CD, CO, NO_2_, and CO remained significantly associated with MetS risk (*p* for trend < 0.02), whereas NO continued to show no significant effect. In Model 3, after adjusting for potential confounders, including shift work, age, marital status, education level, sleep quality, and smoking status, significant positive associations with MetS risk were observed for: CD (Q4: OR = 2.28, 95% CI: 1.60–3.27, *p* < 0.01), CO_2_ (Q4: OR = 1.65, 95% CI: 1.17–2.34, *p* < 0.01), CO (Q4: OR = 1.59, 95% CI: 1.13–2.24, *p* < 0.01), and NO_2_ (Q4: OR = 1.51, 95% CI: 1.08–2.12, *p* = 0.016), NO remained non-significant (*p* for trend = 0.508; Q4: OR = 1.11, 95% CI: 0.79–1.56).

### 3.3. Association Between Mixed Exposure to Underground Air Pollutants and Metabolic Syndrome Risk: BKMR and Qgcomp Models

**Figure 2:**
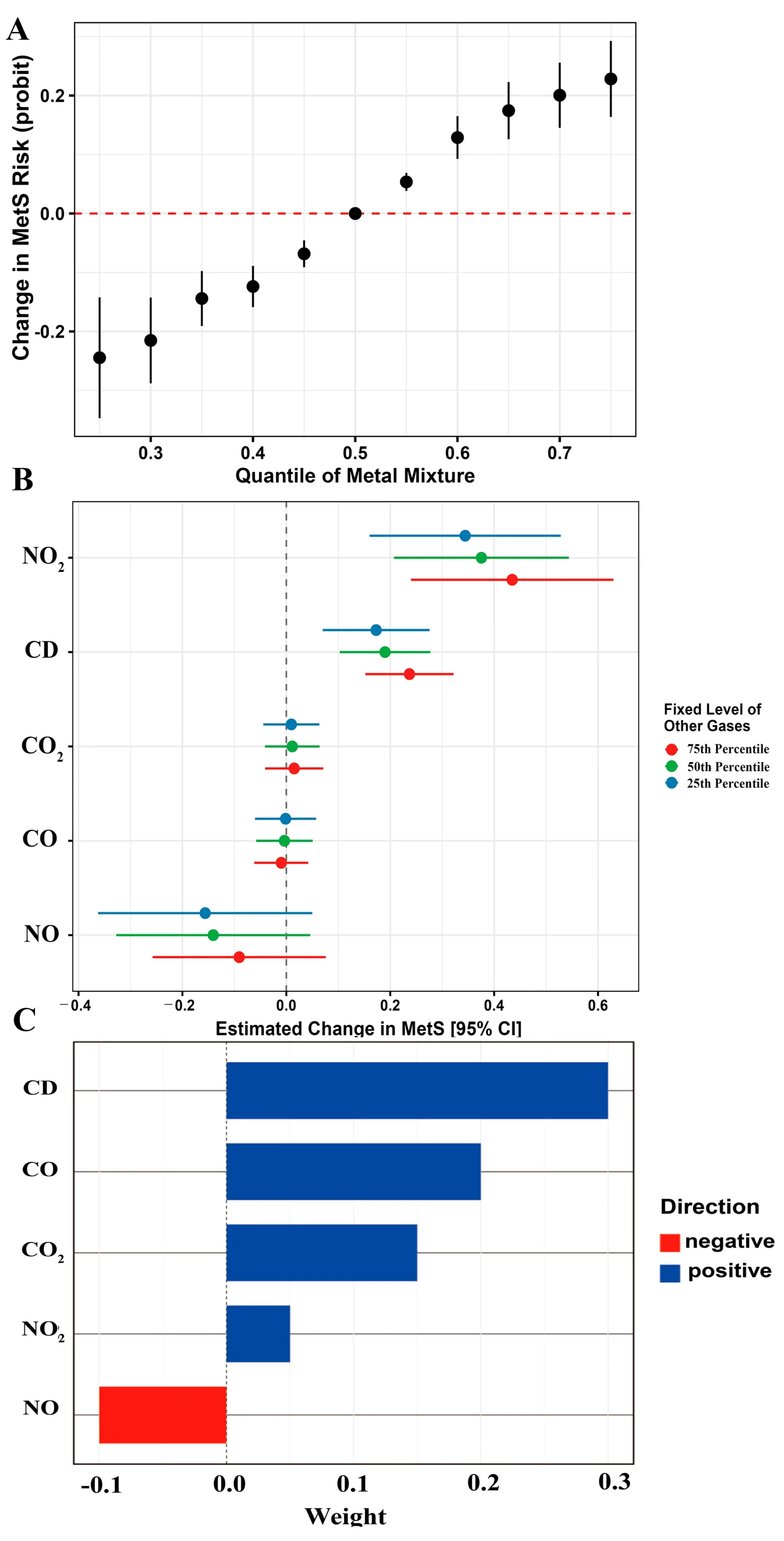
Association between mixed exposure to five underground air pollutants and metabolic syndrome. (**A**) Cumulative effect of mixed pollutant exposure on metabolic syndrome (MetS) risk based on the BKMR model. (**B**) Estimated effects of single pollutants on metabolic syndrome (MetS) risk based on the BKMR model, with other air pollutants held constant. (**C**) Contribution weight analysis of mixed pollutant exposure to metabolic syndrome (MetS) using the Qgcomp model. This model was adjusted for age, smoking, drinking, shift work, education level and sleep quality.

To investigate the potential nonlinear effects and complex interactions among pollutants, we used BKMR models. The findings indicate that exposure to the mixture of pollutants has a positive cumulative toxic effect, leading to a significant increase in the risk of MetS as exposure levels rise (Figure 2A). In addition, CD had the highest positive posterior inclusion probability (PIP) in relation to MetS (Figure S1). Nonlinear exposure–response relationships were observed between the pollutants and MetS, with particularly pronounced increases in risk for CD and NO_2_ at high concentration levels. When the concentrations of the other pollutants were held constant at the 25th, 50th, and 75th percentiles, NO_2_ and CD exhibited significant positive effects, with NO_2_ having a stronger effect than CD (Figure 2B). In contrast, NO did not show statistically significant associations with MetS risk, demonstrating only a slight negative trend. Furthermore, CO_2_ and CO had effects close to zero and were not statistically significant. Additionally, the most pronounced change in the log odds ratio for MetS risk occurred when the exposure levels of CD and NO_2_ increased from the 25th to the 75th percentile. These findings suggest that CD and NO_2_ may have shown the strongest statistical contribution to the increased risk of MetS.

BKMR analyses stratified by metabolic components further revealed distinct pathological pathways (Figures S2 and S3). For the central obesity and hypertension pathways, CD exposure exhibited the strongest and most consistent positive conditional effects on waist circumference and SBP, with the effect curves becoming steeper at higher exposure percentiles. Similar patterns were observed for DBP, suggesting that dust exposure may be an important environmental factor contributing to obesity and hypertension. For disturbances in glucose and lipid metabolism, NO_2_ exposure showed the clearest dose–response relationship with elevated FG and TG levels, following an approximately linear increasing trend. CO and CO_2_ also demonstrated positive associations with TG levels under certain conditions. In addition, CO_2_ exposure emerged as the factor most strongly associated with reduced HDL-C levels in the BKMR model, exhibiting negative conditional effects.

To validate the robustness of the findings, we applied Qgcomp to further explore the weights of individual pollutants in relation to MetS. The results similarly revealed a significant positive association between the mixed exposure index and MetS risk (OR = 1.36, 95% CI: 1.20–1.54; *p* < 0.001). Among the weights contributing to an increased MetS risk, CD had the largest contribution (weight: 0.3), followed by CO (weight: 0.2), whereas NO showed a negative directional weight (weight: −0.1) (Figure 2C). Moreover, analysis of average weights across multiple outcomes further identified CD and NO_2_ as the pollutants with the highest average contributions, consistent with the key findings from the BKMR models (Figure S4). CD and NO_2_ consistently exerted “positive” effects on most metabolic components, including waist circumference, blood pressure, blood glucose, and triglycerides, while displaying a clear negative association with HDL-C. In agreement with previous results, CD exposure showed the strongest positive weight contribution to waist circumference and SBP, with a clearly positive directional effect, reinforcing its central role in driving obesity and hypertension. NO_2_ exposure demonstrated the most prominent positive contribution to FG and TGs, with the largest weights, highlighting its strongest statistical contribution in elevating blood glucose and triglyceride levels.

Two models showed a positive association between mixed exposure to occupational air pollutants and the risk of metabolic syndrome. Among these pollutants, CD and NO_2_ were identified as the most statistical contribution, although they affected the body through different physiological pathways. CD was primarily linked to central obesity and elevated blood pressure, while NO_2_ was more strongly associated with disrupted glucose metabolism and increased triglyceride levels.

### 3.4. Urinary Metabolomics Analysis

Baseline characteristics of participants in the MetS (*n* = 60) and non-MetS (*n* = 60) groups are summarized in Table S1. The two groups showed no significant differences in age, smoking status, alcohol consumption, marital status, sleep quality, or shift work (all *p* > 0.05). However, compared with participants in the non-MetS group, those in the MetS group exhibited significantly greater waist circumference (*p* = 0.001), higher Cd exposure (*p* = 0.004), and higher NO_2_ exposure (*p* = 0.047). In addition, the distribution of education levels differed significantly between the two groups (*p* < 0.001). No significant differences were observed in CO, CO_2_, or NO exposure levels between the groups (all *p* > 0.05).

**Figure 3:**
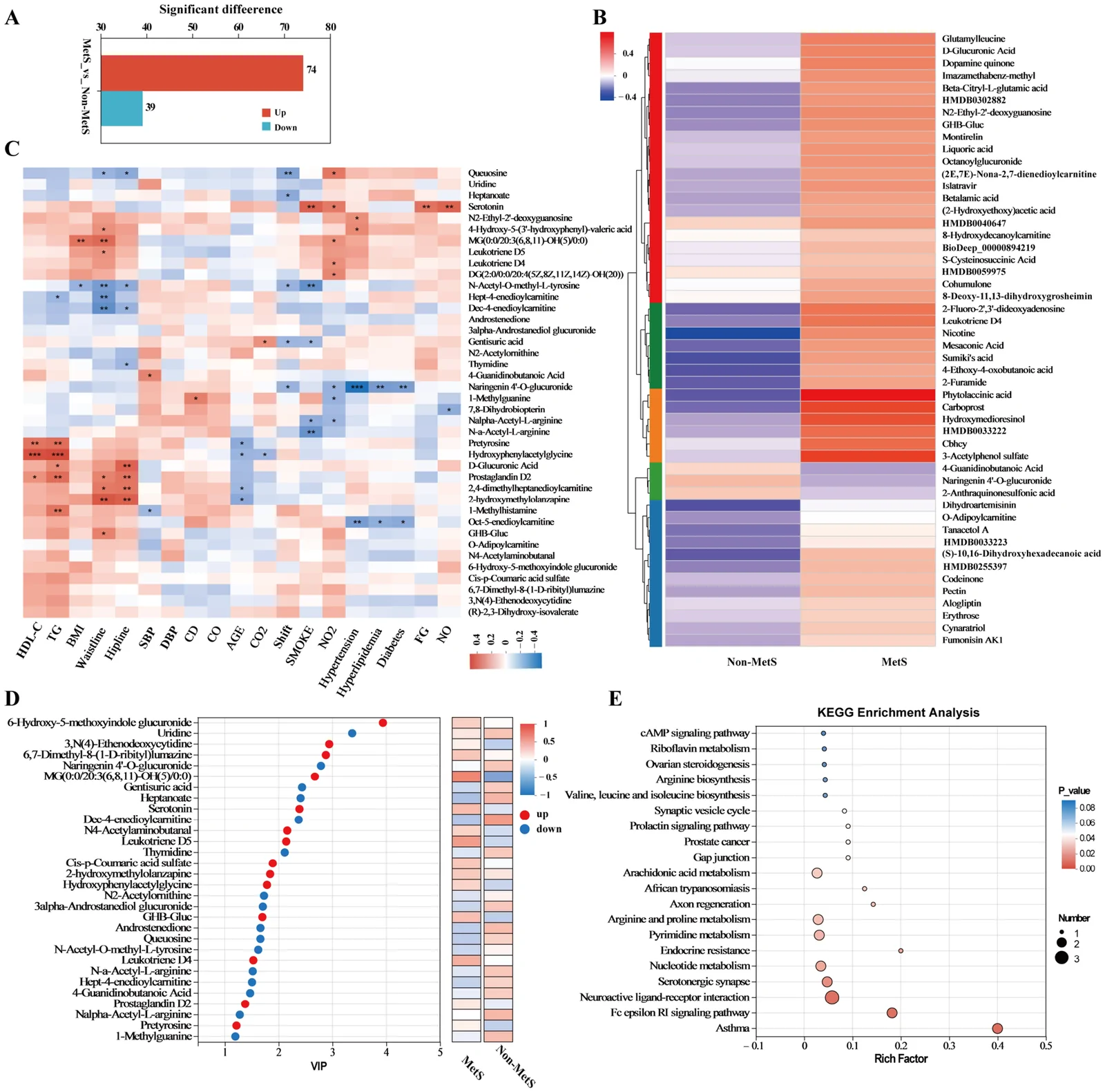
Urinary metabolomics analysis between the MetS and non-MetS groups. (**A**) Number of differentially abundant metabolites. (**B**) Heatmap of 40 endogenous abundant metabolite expression. (**C**) Correlation heatmap of differentially abundant metabolites with metabolic syndrome-related indicators, air pollutants, and lifestyle factors, * indicates *p* < 0.05, ** indicates *p* < 0.01, *** indicates *p* < 0.001. (**D**) VIP ranking plot of differential metabolites; (**E**) KEGG pathway enrichment analysis bubble plot of differentially abundant metabolites.

Among the 120 samples initially analyzed, one MetS sample was excluded due to detection failure, leaving 119 samples for further evaluation. The RSD values of the quality control samples (Pre_QC25 and Raw_QC30) were 0.90% and 0.79%, respectively, demonstrating minimal instrument drift and good analytical reproducibility, thereby supporting the reliability of the dataset (Figure S5A). The partial least squares discriminant analysis (PLS-DA) score plot (Figure S5B) showed a clear separation between the urinary metabolic profiles of the MetS and CK groups along the first principal component, indicating differences in overall endogenous metabolic status. In addition, permutation testing (*n* = 200) was performed to assess the risk of overfitting. The results showed a cumulative R^2^Y of 0.889 (*p* < 0.05) and a Q^2^ of 0.531 (*p* < 0.05), indicating that the model is robust and reliable, with essentially no evidence of overfitting (Figure S5C,D). The differentially abundant metabolites were screened using VIP > 1.0 and FDR q < 0.14. In total, 113 differentially abundant metabolites were identified, of which 74 were upregulated and 39 downregulated (Figure 3A and Table S2). Further analysis revealed 40 endogenous metabolites specifically associated with metabolic syndrome (Figure 3B).

To explore the environmental and clinical relevance of these differentially abundant metabolites, we constructed a correlation network heatmap to visualize the relationships among metabolites, exposure levels, and clinical parameters (Figure 3C). The analysis revealed that several key differential metabolites were significantly associated with major occupational exposure indicators and components of MetS. Specifically, 11 metabolites were linked to air pollutant exposures. Queuosine, Serotonin, MG, Leukotriene D4, DG, Naringenin 4′-O-glucuronide, and Nα-Acetyl-L-arginine showed significant correlations with cumulative NO_2_ exposure. 1-Methylguanine was associated with cumulative CD exposure. Hydroxyphenylacetylglycine correlated with cumulative CO_2_ exposure. 7,8-Dihydrobiopterin was associated with cumulative nitrogen monoxide exposure. Additionally, 17 metabolites were related to metabolic parameters. Pretyrosine, Hydroxyphenylacetylglycine, D-Glucuronic acid, Prostaglandin D2, and 1-Methylhistamine exhibited positive correlations with TG levels, whereas Queuosine, 4-Hydroxy-5-(3′-hydroxyphenyl)-valeric acid, MG, Leukotriene D5, N-Acetyl-O-methyl-L-tyrosine, Hept-4-enedioylcarnitine, Dec-4-enedioylcarnitine, Prostaglandin D2, and 2,4-dimethylheptanedioylcarnitine were associated with waist circumference. Based on variable importance in projection (VIP) values (Figure 3D), the key metabolites contributing most to inter-group differences included uridine, serotonin, heptanoate, leukotriene D4/D5, thymidine, prostaglandin D2, as well as several acylcarnitines (e.g., Dec-4-enedioylcarnitine) and amino acid derivatives (e.g., N-alpha-acetylarginine).

KEGG pathway enrichment analysis showed that the differential metabolites mentioned earlier were significantly enriched in several key metabolic pathways, including pyrimidine metabolism, phenylalanine metabolism, arachidonic acid metabolism, and primary bile acid biosynthesis (Figure 3E). These pathways play essential roles in nucleotide homeostasis, the generation of inflammatory mediators, neurotransmitter regulation, and lipid digestion and absorption—metabolic networks that could be disrupted by exposure to occupational pollutants.

## 4. Discussion

Airborne pollutants in underground coal mines are typically present at high concentrations, exhibit complex chemical compositions, and result in prolonged occupational exposure [37]. Coal miners chronically exposed to these pollutants face an elevated risk of multiple adverse health outcomes. While previous research has primarily focused on respiratory effects [39], accumulating evidence indicates that long-term inhalation of CD and NO_x_ may disrupt metabolic homeostasis, potentially contributing to both the development and progression of MetS [38,40,41]. Epidemiological studies consistently report higher prevalences of dyslipidemia, hyperglycemia, and hypertension among coal miners compared with non-dust-exposed occupational groups or general population controls. Furthermore, environmental epidemiological investigations have associated elevated ambient PM_2.5_ concentrations with increased MetS prevalence [13]. Collectively, these findings suggest that dust exposure is not only a well-established respiratory hazard but also a plausible risk factor for systemic metabolic dysfunction.

In the present study, we comprehensively evaluated the associations between occupational exposure to five major underground air pollutants (CD, CO, CO_2_, NO_2_, and NO) and MetS risk in a cohort of coal miners. We further explored underlying molecular mechanisms using untargeted urinary metabolomics. In single-pollutant models, cumulative occupational exposures to CD, CO, CO_2_, and NO_2_ were positively associated with increased MetS risk, whereas NO exposure showed no statistically significant association. Mixture analyses using BKMR and Qgcomp jointly identified CD and NO_2_ as the predominant pollutants associated with MetS risk. However, their pathophysiological profiles differed markedly. CD exposure was most strongly linked to central obesity and elevated blood pressure, whereas NO_2_ exposure was preferentially associated with hyperglycemia and hypertriglyceridemia. Notably, although CO_2_ exposure showed an inverse association with HDL-C in the single-pollutant model, this effect attenuated to near-null in the mixture models, precluding a direct causal interpretation. Elevated CO_2_ in mining environments primarily reflects poor ventilation and blasting intensity, conditions under which CD and NO_2_ also accumulate. Thus, CO_2_ likely serves as a surrogate marker of hazardous exposure microenvironments rather than a direct determinant of HDL-C. Nevertheless, emerging evidence suggests that environmentally relevant CO_2_ elevations may induce oxidative stress, systemic inflammation, and endothelial dysfunction—biological pathways that could potentially affect lipoprotein metabolism [42,43]. Future studies are warranted to clarify its independent effects. Untargeted urinary metabolomics revealed exposure-dependent perturbations across multiple metabolic pathways, including pyrimidine metabolism, phenylalanine metabolism, arachidonic acid metabolism, and primary bile acid biosynthesis—indicating systemic metabolic dysregulation.

In the single-pollutant analysis, cumulative exposure to CD was significantly and positively associated with MetS risk. Participants in the highest quartile (Q4) had twice the odds of MetS compared with those in the lowest quartile (OR = 2.28, 95% CI: 1.60–3.27, *p* < 0.01). CD also exhibited the highest PIP in the BKMR model, further suggesting that it is the pollutant most strongly associated with metabolic alterations among those studied. This finding aligns with existing epidemiological evidence. Multiple studies have reported significant associations between long-term exposure to inhalable particulate matter and key features of MetS, such as insulin resistance, central obesity, and dyslipidemia [44]. Notably, a large longitudinal cohort study in Taiwan, including nearly 94,000 adults, found that long-term PM_2.5_ exposure was independently associated with increased risk of MetS and each of its five diagnostic components (abdominal obesity, elevated triglycerides, reduced HDL cholesterol, hypertension, and hyperglycemia). Moreover, co-exposure to PM_2.5_ and NO_2_ further amplified MetS risk, particularly among individuals with pre-existing metabolic abnormalities (e.g., prediabetes or hypertension) [18]. Mechanistically, inhaled fine particles can induce systemic oxidative stress and low-grade chronic inflammation, promoting the release of pro-inflammatory cytokines such as TNF-α and IL-6, which in turn interfere with insulin signaling [45]. In addition, CD carries toxic chemical constituents including heavy metals and polycyclic aromatic hydrocarbons, which can induce oxidative stress and directly impair mitochondrial electron transport chain function, leading to impaired fatty acid β-oxidation and reduced energy metabolic efficiency [46]. The metabolic consequences of mitochondrial dysfunction include accumulation of lipid intermediates and ectopic lipid deposition, particularly prominent in abdominal visceral adipose tissue, which is a key driver of central obesity. Concurrently, mitochondrial dysfunction can activate the renin-angiotensin system and enhance sympathetic tone, thereby promoting elevated blood pressure [47,48]. In the present study, CD showed the largest weight contribution to waist circumference and systolic blood pressure, further supporting the hypothesis that dust exposure affects MetS through obesity- and blood pressure-related pathways. Of note, the chemical composition of CD in underground coal mines is extremely complex and differs fundamentally from that of atmospheric PM_2.5_, comprising coal dust, rock dust, and silica among other mineral particles [38]. The biological effects of these components may differ substantially—a limitation that cannot be fully captured by a single cumulative exposure metric.

In addition, NO_2_ exposure was significantly and positively associated with MetS risk (Q4: OR = 1.51, 95% CI: 1.08–2.12) and showed the strongest effects on blood glucose and triglycerides in the BKMR model, suggesting that NO_2_ may primarily contribute to MetS through disruption of glucose and lipid metabolism. Experimental studies have confirmed that NO_2_ exposure impairs glucose metabolism by reducing glycogen synthesis and increasing gluconeogenesis, while also promoting lipid deposition through enhanced lipogenesis and uptake, ultimately increasing lipid oxidation and secretion [49,50]. Population-based studies have further demonstrated that NO_2_ exposure is significantly associated with elevated fasting glucose and increased risk of type 2 diabetes [50,51]. In addition, a meta-analysis reported that each 10 μg/m^3^ increase in ambient NO_2_ was associated with a 4.24% (1.37–7.19%) increase in triglyceride levels [52]. As a potent oxidizing gas, NO_2_ can induce lipid peroxidation and oxidative modification of very-low-density lipoprotein, thereby impairing triglyceride clearance [53,54]. In the present study, NO_2_ carried the largest positive weight for triglycerides, aligning with the mechanistic inferences above. In contrast, NO showed no significant association in either the single-pollutant or mixture models. This may be attributable to its relatively low concentration in the underground coal mine environment (median: 1.29–1.35 mg/m^3^-year), which falls below the known biological threshold for adverse effects. Alternatively, it may reflect the fact that NO, as a short-lived signaling molecule, is poorly captured by cumulative exposure metrics that cannot adequately account for its rapid metabolism and signaling kinetics [55].

The BKMR and qgcomp models mutually corroborated each other, consistently identifying Cd and NO_2_ as the dominant components contributing most strongly to the observed associations, although they may act through distinct pathological pathways. Specifically, CD exhibited the strongest effects on waist circumference and systolic blood pressure, whereas NO_2_ showed the greatest effects on fasting glucose and triglycerides. This pattern of pollutant–component specificity carries important public health implications: when establishing occupational exposure limits and intervention strategies, it is essential to consider not only the overall effect of the mixture but also the differential health impacts of individual pollutants. For example, combined control of CD and NO_2_ may yield synergistic benefits for MetS prevention, whereas targeted reduction of CO_2_ emissions—likely reflecting improved ventilation rather than a direct toxicological benefit—may hold value in reducing overall hazardous exposure. Mechanistically, CD particles are relatively large and tend to deposit in the alveoli and bronchioles, where they induce local inflammation that subsequently affects systemic metabolism [38]. In contrast, NO_2_ is a water-soluble gas that is readily absorbed in the upper respiratory tract and rapidly enters the circulation, potentially acting directly on metabolic organs such as the liver and pancreas [53]. Notably, although CO and CO_2_ were significantly associated with MetS in the single-pollutant models, their effects approached null and were not statistically significant in the mixture models. This suggests that their impacts may be masked by CD- and NO_2_-dominated pathological processes, or that they serve more as collinear exposure surrogates rather than direct causal agents. Given the cross-sectional design of the present study, these causal inferences require further validation through longitudinal investigations. It is also essential to clarify the conceptual distinction between the “mixture exposure” examined in this study and the actual airborne mixture inhaled by workers. In our analysis, the term “mixed exposure” refers to the statistical joint effect of five pollutants estimated through BKMR and Qgcomp models, where each pollutant’s contribution is weighted based on its conditional association with MetS risk—rather than reflecting a physical or toxicological interaction among the pollutants.

Urinary metabolomics provided a partial molecular-level explanation for the observed exposure–disease associations. In MetS patients with high pollutant exposure, multiple metabolic pathways were found to be perturbed. Disturbances in pyrimidine metabolism were indicative of imbalances in nucleotide synthesis and energy homeostasis [56]. Phenylalanine metabolism alterations have been linked to insulin resistance and endothelial dysfunction [57]. Arachidonic acid metabolism is a major source of inflammatory mediators, including prostaglandins and leukotrienes, and is directly associated with obesity-related chronic inflammation [58]. Several differentially abundant metabolites showed significant correlations with cumulative NO_2_ exposure, including serotonin, leukotriene D4, and Nα-acetylarginine, supporting the notion that NO_2_ affects metabolism through inflammatory pathways [59]. In addition, multiple acylcarnitines associated with waist circumference (e.g., 4-hydroxy-5-(3′-hydroxyphenyl)-valerylcarnitine and dec-4-enedioylcarnitine) are established biomarkers of incomplete mitochondrial fatty acid oxidation, suggesting that CD and NO_2_ exposure may interfere with mitochondrial function and promote ectopic lipid deposition [60,61]. These metabolomic findings corroborate the results of the epidemiological models and provide new insights into the mechanisms underlying pollutant-induced metabolic disruption. Nevertheless, it should be noted that the metabolomics sample size in the present study was relatively small, which may have limited the statistical power to detect subtle metabolic alterations. Therefore, the candidate molecules identified through this screening require validation in larger targeted cohorts.

## 5. Conclusions

In this study, by integrating epidemiological and metabolomic approaches, we systematically demonstrated that mixed exposure to underground air pollutants is significantly associated with MetS risk among coal miners. Mixed exposure to inhalable dust and nitrogen dioxide represents an important occupational risk factor for metabolic syndrome. Specifically, CD was primarily associated with central obesity and elevated blood pressure, whereas NO_2_ was closely related to glucose dysregulation and elevated triglycerides. The disturbances in pyrimidine metabolism, arachidonic acid metabolism, and bile acid metabolism revealed by urinary metabolomics may contribute to the molecular mechanisms underlying these associations. These findings underscore the importance of simultaneously controlling exposure to multiple air pollutants in occupational populations and provide a scientific basis for developing differentiated health surveillance strategies. Future research should incorporate refined pollutant component analysis and dynamic metabolomics monitoring within prospective cohort designs to further elucidate the causal pathways from exposure to metabolism to disease.

## 6. Advantages and Limitations

This study has several strengths. First, we employed an analytical strategy that combined single-pollutant models with two mixture models (BKMR and Qgcomp), which allowed us both to identify key toxic components and to characterize the overall mixture effect. The consistency of the results across the two mixture models enhanced the robustness of our conclusions. Second, we integrated epidemiological analyses with urinary metabolomics, extending the investigation from exposure–disease associations to the molecular level and providing independent data to support mechanistic inferences. Third, our exposure assessment was based on detailed work-history records and cumulative exposure calculations, which helped minimize exposure misclassification bias.

Several limitations should also be acknowledged. The cross-sectional design precludes establishing temporal relationships between exposure and MetS; therefore, causal inference requires validation in prospective cohort studies. Cumulative exposure estimates were reconstructed retrospectively from historical monitoring data, which may have introduced uncertainty, particularly for early-year exposures. Nonetheless, non-differential misclassification typically biases effect estimates toward the null, suggesting that the significant associations observed in this study may be conservative. In addition, the relatively small sample size of the metabolomics analysis may have limited the statistical power to detect subtle metabolic alterations. Therefore, the candidate molecules identified in this study should be further validated in larger targeted cohorts. Finally, MetS was defined according to the 2009 joint interim criteria (with Asian-specific waist circumference cutoffs). The use of different diagnostic criteria could affect prevalence estimates and comparability with other studies.

## Figures and Tables

**Table 1:** Baseline clinical characteristics of coal miners in the MetS and Non-MetS groups.

Characteristics	Non-MetS (*n* = 957)	MetS (*n* = 457)	t/χ^2^	*p* Value
Age, years, mean ± SD	38.80 ± 7.19	40.67 ± 7.59	−4.498	<0.001
Waist Circumference, cm, mean ± SD	84.65 ± 8.39	93.91 ± 7.01	−20.398	<0.001
Height, cm, mean ± SD	172.20 ± 5.84	170.99 ± 5.87	3.63	<0.001
Weight, kg/m^2^, mean ± SD	75.36 ± 11.84	75.01 ± 10.95	0.543	0.587
CD, mg/m^3^-years, M (P25, P75)	21.88 (17.18, 28.11)	24.99 (19.55, 38.28)	−7.123	<0.001
CO, mg/m^3^-years, M (P25, P75)	26.94 (19.38, 45.92)	29.64 (21.76, 51.62)	1.412	0.158
CO_2_, mg/m^3^-years, M (P25, P75)	24,429.26 (13,262.29, 31,579.86)	26,164.77 (18,400.70, 34,842.28)	−4.43	<0.001
NO, mg/m^3^-years, M (P25, P75)	1.29 (0.45, 1.79)	1.35 (0.57, 1.89)	−0.927	0.354
NO_2_, mg/m^3^-years, M (P25, P75)	2.05 (0.45, 5.01)	3.67 (0.51, 5.73)	−4.725	<0.001
Education level			57.943	<0.001
Below high school	24 (2.5%)	19 (4.2%)		
High school or equivalent	253 (24.6%)	208 (45.5%)		
Above high school	680 (71.1%)	230 (50.3%)		
Shift work			15.242	<0.001
No	365 (38.1%)	126 (27.6%)		
Yes	592 (61.9%)	331 (72.4%)		
Hypertension			27.082	<0.001
No	875 (91.4%)	379 (82.9%)		
Yes	57 (6%)	65 (14.2%)		
Unspecified	25 (2.6%)	13 (2.8%)		
Hyperlipidemia			13.782	0.001
No	882 (92.2%)	399 (87.3%)		
Yes	48 (5%)	47 (10.3%)		
Unspecified	27 (2.8%)	11 (2.4%)		
Diabetes			7.352	0.025
No	920 (96.1%)	429 (93.9%)		
Yes	14 (1.5%)	17 (3.7%)		
Unspecified	23 (2.4%)	11 (2.4%)		
Smoking			0.01	0.92
Never	368 (38.5%)	177 (38.7%)		0.483
Current or former smokers	589 (61.5%)	280 (61.3%)		
Alcohol consumption			0.324	0.569
Never	485 (50.7%)	239 (52.3%)		
Current or former drinking	472 (49.3%)	218 (47.7%)		
Marital status			8.527	0.003
Never married	86 (9.0%)	21 (4.6%)		
Married	871 (91.0%)	436 (95.4%)		
Sleep quality			5.422	0.247
Good	358 (37.4%)	193 (42.2%)		
Fair	417 (43.6%)	174 (38.1%)		
Poor	151 (15.8%)	72 (15.8%)		
Very poor (with medication assistance)	13 (1.4%)	10 (2.2%)		
Unspecified	18 (1.9%)	8 (1.8%)		

## Data Availability

The raw data supporting the conclusions of this article will be made available by the authors on request.

## Supplementary Materials

The following supporting information can be downloaded at: https://www.mdpi.com/article/10.3390/toxics14090746/s1, Figure S1. PIP results for the five pollutants in the BKMR model; Figure S2. Cumulative effects of mixed exposure on the risk of individual MetS components based on the BKMR model. The x-axis represents the quantile of the pollutant mixture, and the y-axis represents the change in risk (probit scale) of each metabolic component relative to the median exposure level (set to 0, red dashed line). Black dots denote posterior mean estimates at each exposure quantile, and black vertical lines represent 95% confidence intervals; Figure S3. Association between mixed exposure levels of pollutants and the risk of individual metabolic syndrome components based on the BKMR model. The x-axis represents the quantile of the pollutant mixture, and the y-axis represents the change in risk (probit scale) of each metabolic component. The red dashed line is the reference line at an effect of zero. Points and error bars indicate the estimated effect sizes and 95% confidence intervals, respectively; Figure S4. Contribution weights of air pollutants to individual metabolic syndrome components. The x-axis represents the weight coefficient of each pollutant. Blue bars indicate a positive effect (positive weight), red bars indicate a negative effect (negative weight), and the length of each bar represents the magnitude of the pollutant’s contribution to the corresponding component; Figure S5. Quality control assessment, PLS-DA score plot, and permutation test for urinary metabolomic profiles of the MetS and non-MetS groups. (A) RSD values of QC samples; (B) PLS-DA score plot showing group separation; (C,D) Permutation test results demonstrating model robustness; Table S1. Baseline characteristics of MetS and Non-MetS groups; Table S2. The differentially abundant metabolites of MetS and Non-MetS groups.

## Abbreviations

The following abbreviations are used in this manuscript:

MetS	Metabolic syndrome
BKMR	Bayesian kernel machine regression
Qgcomp	quantile-based g-computation
CD	coal dust
CO	carbon monoxide
CO_2_	carbon dioxide
NO_2_	nitrogen dioxide
NO	Nitric oxide
FG	fasting glucose
TGs	triglycerides
HDL-C	high-density lipoprotein cholesterol
SBP	systolic blood pressure
DBP	diastolic blood pressure
WC	waist circumference
PLS-DA	Partial least squares discriminant analysis
VIP	variable importance in projection
KEGG	Kyoto Encyclopedia of Genes and Genomes
